# Animal Diseases Caused by Orbiviruses, Algeria

**DOI:** 10.3201/eid1712.110928

**Published:** 2011-12

**Authors:** Hafsa Madani, Jordi Casal, Anna Alba, Alberto Allepuz, Catherine Cêtre-Sossah, Leila Hafsi, Houria Kount-Chareb, Nadera Bouayed-Chaouach, Hassiba Saadaoui, Sebastian Napp

**Affiliations:** Institut National de Médecine Vétérinaire, Algiers, Algeria (H. Madani, L. Hafsi, H. Kount-Chareb, N. Bouayed-Chaouach, H. Saadaoui);; Centre de Recerca en Sanitat Animal, Barcelona, Spain (J. Casal, A. Alba, A. Allepuz, S. Napp);; Universitat Autònoma de Barcelona, Barcelona (J. Casal, A. Allepuz);; Centre de Coopération Internationale en Recherche Agronomique pour le Développement, Montpellier, France (C. Cetre-Sossah)

**Keywords:** bluetongue virus, epizootic hemorrhagic disease virus, African horse sickness virus, orbiviruses, viruses, zoonoses, animal diseases, livestock, cattle, sheep, goats, camels, antibodies, Algeria

## Abstract

Antibodies against bluetongue virus were detected in cattle, sheep, goats, and camels in Algeria in 2008. Antibodies against epizootic hemorrhagic disease virus were detected in cattle, but antibodies against African horse sickness virus were not detected in horses and mules. Epizootic hemorrhagic disease in northern Africa poses a major risk for the European Union.

The genus *Orbivirus* contains several viruses such as bluetongue virus (BTV; 26 serotypes), epizootic hemorrhagic disease virus (EHDV; 8 serotypes), and African horse sickness virus (AHSV; 9 serotypes). These viruses cause serious diseases in domestic and wild animals. Several orbiviruses have been reported in Algeria. Bluetongue disease caused by BTV serotype 2 (BTV-2) was detected in 2000 (*1*); serotype 1 was detected in 2006 (*2*). BTV-1 was detected again in 2008, and new outbreaks were detected in 2009 and 2010 (*2*). Epizootic hemorrhagic disease (EHD) caused by EHDV serotype 6 (EHDV-6), was reported in Morocco in 2004 and 2006 (*3*). AHSV serotype 9 (AHSV-9) was detected in Morocco in 1965 (*4*).

These orbiviruses were frequently reported to have originated in sub-Saharan Africa (*3**,**5**–**7*). In many cases, outbreaks of orbivirus diseases in Algeria were followed by incursions of these viruses into southern Europe, most likely through passive wind-borne transmission of infected vectors (*8**,**9*). The aim of this study was to identify 3 particular orbiviruses (BTV, EHDV, and AHSV) in Algeria and determine their geographic distribution.

## The Study

Algeria contains 19.6 million sheep, 3.8 million goats, 1.6 million cattle, ≈230,000 horses, and 290,000 camels. This country is divided into 48 provinces (wilayas). For reasons of animal health, transportation of animals is not allowed between southern and northern Algeria. Vaccination against bluetongue disease, EHD, and African horse sickness in Algeria is forbidden by law because vaccinated animals cannot be differentiated from naturally infected animals.

Sampling was conducted during August–September 2008. Cattle, sheep, goats, and camels were sampled in the BTV survey, cattle were sampled in the EHDV survey, and horses and mules were sampled in the AHSV survey. To avoid detection of antibodies from previous outbreaks, only livestock 6–12 months of age were sampled. For detection of EHDV and BTV, the epidemiologic unit was the herd.

Sample size was calculated to enable detection of >2% of infected cattle farms at a 95% confidence level (149 herds) and a within-herd prevalence >30% (9 animals/herd). In addition to cattle for detection of BTV, 359 samples were obtained from 65 sheep flocks, 71 samples from 27 goat herds, and 92 samples from 26 camel herds. For detection of AHSV, the epidemiologic unit was the animal, and sample size was calculated for detection of >2% of infected horses and mules at a 95% confidence level (149 animals).

IgG against BTV was detected by using a competitive ELISA (Pourquier Bluetongue Competitive ELISA; Pourquier Laboratory, Montpellier, France). To detect BTV genotypes, a real-time reverse transcription PCR (RT-PCR) (TaqVet BTV-FCO-all genotypes rRT-PCR; LSI Vet, Lissieu, France) was performed. Positive samples were tested by using RT-PCR kits for BTV-1, 2, 4, 6, 9, 11, and 16 (Taqvet BTV European BTV Typing; LSI Vet). A competitive ELISA provided by the Institute of Animal Health (Pirbright, UK) was performed to detect IgG against EHDV according to the protocol of Thevasagayam et al. (*10*). IgG against AHSV was detected by using a blocking ELISA (Ingezim AHSV compact plus 14.AHS.K.3; Ingenasa Laboratory, Madrid, Spain). Tests were performed according to manufacturer’s instructions.

We detected an overall BTV seroprevalence of 24%. BTV seroprevalence differed among wilayas (Table; Figure) but was higher in northern wilayas and Ghardaia in central Algeria. In contrast with official 2008 data in which only 6 outbreaks were reported, our results indicated that BTV was widespread in 2008.

**Table:** Seropositivity for BTV and EHDV in livestock in Algeria, 2008*

Location†	No. positive/no. tested				
	BTV, cattle	BTV, sheep	BTV, goats	BTV, camels	EHDV, cattle
Adrar	0/31	0/19	0/6	0/27	6/29
Aïn Témouchent (1)	13/54	1/16	1/11	NT	1/54
Algiers (2)	14/41	0/18	NT	NT	0/41
Annaba (3)	8/28	1/17	NT	NT	0/28
Béjaïa (4)	6/40	0/21	NT	NT	0/40
Blida (5)	13/44	4/25	NT	NT	1/44
Boumerdès (6)	12/37	3/15	NT	NT	1/37
Chlef (7)	12/22	0/21	NT	NT	1/22
Djelfa	5/49	2/13	0/5	NT	15/49
El Tarf (8)	54/54	9/17	NT	NT	3/54
Ghardaia	17/22	6/15	5/12	7/7	5/22
Jijel (9)	4/27	0/22	NT	NT	0/27
Mostaganem (10)	58/72	NT	NT	NT	11/72
Naama (11)	0/55	2/23	0/4	10/35	9/56
Oran (12)	9/53	7/18	NT	NT	11/53
Skikda (14)	3/13	3/11	NT	NT	1/13
Souk Ahras (15)	8/18	0/9	NT	NT	1/18
Tebessa (16)	2/26	6/17	4/11	NT	6/26
Tindouf		6/15	5/13	2/23	
Tipasa (16)	0/48	0/20	NT	NT	0/48
Tizi Ouzou (17)	0/64	1/9	NT	NT	0/62
Tlemcen (18)	9/54	1/18	0/9	NT	3/54
Total	247/852	52/359	15/71	19/92	75/849

*BTV, bluetongue viruses; EHDV, epizootic hemorrhagic disease virus; NT, not tested. †Values in parentheses indicate provinces (wilayas) shown in the Figure.

**Figure:**
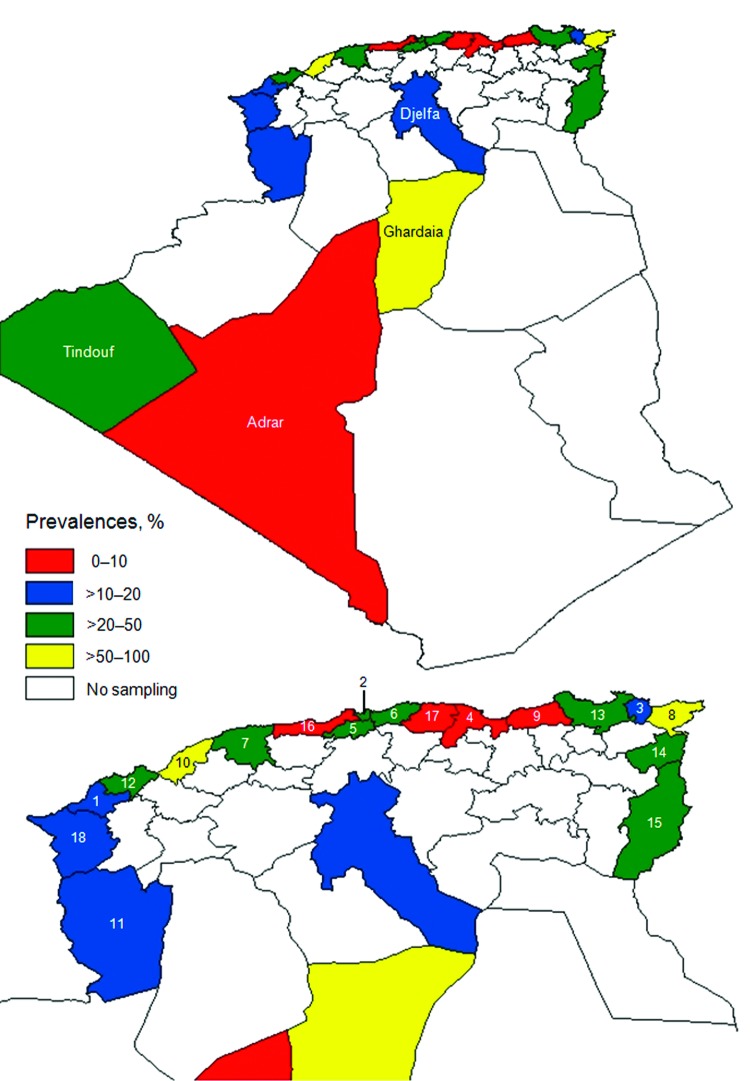
Seroprevalence of bluetongue virus in cattle, sheep, goats, and camels, by province (wilaya), Algeria, 2008. A) Entire country; B) Northern Algeria. 1, Aïn Témouchent; 2, Algiers; 3, Annaba; 4, Béjaïa; 5, Blida; 6, Boumerdès; 7, Chlef; 8, El Tarf; 9, Jijel; 10, Mostaganem; 11, Naama; 12, Oran; 13, Skikda; 14, Souk Ahras; 15, Tébessa; 16, Tipasa; 17, Tizi Ouzou; 18, Tlemcem.

BTV seroprevalence differed between species (Table): 29% in cattle, 14% in sheep, and 21% in goats. In addition, a high seroprevalence (21%) was found in camels. In a recent study, BTV was isolated from the blood of 3 experimentally infected camels, which indicated that this animal might play a role in BTV transmission (*11*). Given that camels are frequently moved across desert areas in Algeria, they could potentially transport BTV over long distances, enabling viruses to cross the Sahara Desert.

Of ELISA-positive samples, 335 samples (250 from cattle, 51 from sheep, 15 from goats, and 19 from camels) obtained in 20 wilayas throughout Algeria were tested by BTV RT-PCR. BTV RNA, which indicates recent infection, was detected in 37 of samples (34 from cattle and 3 from sheep), most of which had been obtained in northeastern Algeria. The serotype identified was BTV-1.

Antibodies against EHDV were detected in 9% of the cattle tested. EHDV seroprevalence was detected in 15 of 21 wilayas sampled (Table), although EHD was not officially reported in 2008. This finding might be explained by often inconclusive clinical diagnoses and the factthat definitive diagnoses of this disease require specific laboratory tests (*3*). Given that only animals 6–12 months of age were sampled, seropositivity indicated circulation of EHDV over the previous year; the last reported outbreak in Algeria was in September 2006. In addition, the EHD epidemic in 2006 affected only central Algeria. However, our results indicate that EHD was widespread in 2008. EHDV seroprevalence seemed to be higher in central and southern Algeria (Table). None of 145 mules and 6 horses sampled in southwestern Algeria had antibodies against AHSV.

## Conclusions

Our results indicated that BTV and EHDV were widespread in Algeria. Distribution of orbiviruses is determined by distribution of competent vectors, and entomologic surveys indicated that *Culicoides imicola* midges are abundant in northern and central Algeria (*12*), which is consistent with our results. Conversely, *C. imicola* midges were not present in southern desert regions, which would indicate that the livestock were infected elsewhere or that other *Culicoides* spp. might play a role in transmission. Moreover, although BTV seroprevalence was higher in northern and central wilayas, EHDV seroprevalence was higher in southern and central regions. This finding might be explained by the fact that different vector species can transmit EHDV and BTV (*3*). The temperature requirements for replication of these viruses and further transmission are also likely to differ (*3*). Furthermore, differences in distribution of these viruses might be influenced by exposure to viruses in previous years (*13*).

EHDV in northern Africa poses a major risk for the European Union because of likely wind-borne dispersal of infected vectors (*3*). In Europe, the presence of a known competent vector for EHDV (*C. imicola*), plus several suspected vectors (*14*), and the climatic conditions could be conducive to EHDV circulation (*3*). As reported in Israel in 2006, an EHDV epidemic can have a major economic effect through loss of milk production and increased animal deaths (*15*). If EHDV were introduced into the European Union, detection of infected animals would be hampered by a lack of diagnostic methods (*3*). EHD control would also be complicated by a lack of vaccines (*3*). In addition, the high prevalence of BTV in camels in Algeria and their potential role in BTV transmission warrants further investigation.
